# Association between triglyceride-glucose index and intracranial/extracranial atherosclerotic stenosis: findings from a retrospective study

**DOI:** 10.1186/s12933-024-02187-1

**Published:** 2024-03-14

**Authors:** Yu Xie, Kuan Cen, Bitang Dan, Li Zou, Lei Zhang, Renwei Zhang, Huagang Li, Qi Cai, Nadire Aiziretiaili, Zhenxing Liu, Yumin Liu

**Affiliations:** 1 Department of Neurology, Zhongnan Hospital of Wuhan University, 169 Donghu Road, Wuhan, 430000 Hubei China; 2 Department of Neurology, The First People’s Hospital of Kashi Prefecture, Kashi, 844000 China; 3 Department of Neurology, Yiling Hospital of Yichang City, Yichang, 443000 Hubei China

**Keywords:** Triglyceride-glucose index, Intracranial atherosclerotic stenosis, Extracranial atherosclerotic stenosis

## Abstract

**Objective:**

The association of the triglyceride-glucose (TyG) index with intracranial atherosclerotic stenosis (ICAS) and extracranial atherosclerotic stenosis (ECAS) is unclear. This study aimed to investigate the relationship of TyG index with the distribution and severity of ICAS and ECAS.

**Method:**

Patients who underwent digital subtraction angiography (DSA) for evaluating ICAS/ECAS in Zhongnan Hospital of Wuhan University from January 2017 to October 2021 were retrospectively enrolled in our study. Clinical characteristics, DSA data, blood routine, lipid profile and fasting glucose were recorded. The association of TyG index and ICAS/ECAS status were investigated in four aspects: location and distribution of stenosis, stenosis severity and whether stenosis is symptomatic. Logistic regression models were used to evaluate the association. Restricted cubic splines were constructed to model the non-linear relationship between the TyG index and different arterial stenosis status.

**Results:**

Among 1129 included patients, the median age was 62 (IQR 55–68) years, and 71.3% were male. The median TyG index was 8.81 (8.40, 9.21). Elevated TyG index was significantly associated with ICAS, combined ICAS/ECAS, anterior circulation stenosis, posterior circulation stenosis, combined anterior/posterior circulation stenosis, severe stenosis, both asymptomatic and symptomatic stenosis. This association was maintained after adjusting for age, sex, smoking, drinking, medical history of hypertension and stroke, platelet, total cholesterol, high-density lipoprotein, and low-density lipoprotein. Multivariable-adjusted spline regression models showed that a progressively increasing risk of arterial stenosis was related to an elevated TyG index.

**Conclusion:**

Elevated TyG index was associated with ICAS/ECAS. TyG index might be a useful indicator of ICAS and severe stenosis.

## Background

Stroke remains a leading cause of death and disability globally, with ischemic stroke representing the majority of stroke types [1]. Intracranial and extracranial atherosclerotic stenosis (ICAS and ECAS) is main cause of ischemic stroke [2, 3]. It is of great importance to identify biomarkers of artery stenosis as early as possible to prevent ischemic stroke.

Triglyceride-glucose (TyG) index, calculated by fasting blood glucose and triglyceride, was proposed as a substitute marker of insulin resistance. It has been suggested to be associated with cardiovascular and cerebrovascular diseases [4–8]. In an American population with diabetes or pre-diabetes, the baseline TyG index was associated with cardiovascular disease and all-cause mortality [4]. TyG index was also proposed as an independent indicator for predicting the severity and prognosis of multivessel coronary disease in patients with acute coronary syndrome [5]. In the field of cerebrovascular disease, elevated TyG index has been suggested to be an independent predictor of ischemic stroke and could identify ischemic stroke patients at high risk of death [6, 7]. Elevated TyG index was also associated with increased odds of ICAS and ECAS [8]. As we know, evidence has indicated that the prevalence of risk factors differs between ICAS and ECAS [9]. ICAS seems to be more closely associated with metabolic derangement [9, 10]. Therefore, the investigation of the association of TyG index with ICAS and ECAS could be interesting. Furthermore, the arterial stenosis in the previous studies was assessed by computed tomography angiography (CTA), magnetic resonance angiography (MRA) or ultrasonography. Few studies have evaluated vascular stenosis by digital subtraction angiography (DSA), which is the current standard for diagnosing intracranial and extracranial stenosis.

In our study, we aimed to investigate the association of TyG index and the severity and distribution of cerebrovascular atherosclerotic stenosis, which was evaluated by DSA. It will be helpful for the early identification and diagnosis of cerebrovascular stenosis.

## Subjects and methods

This retrospective clinical research was conducted at a single center. Patient selection, clinical/imaging data collection and analysis process has been published previously [11]. 

### Patient selection

In our center, when patients suspect cerebral arterial stenosis, DSA examination will be performed with the patient’s consent. We enrolled consecutive middle-aged and elderly patients who underwent cerebrovascular DSA examination in the department of neurology in Zhongnan Hospital of Wuhan University from January 2017 to October 2021. Patients with arterial stenosis caused by reasons other than atherosclerosis were excluded. The exclusion criteria were as follows: non-Chinese nationality; younger than 45-year-old; incomplete DSA data or laboratory tests; evidence of cardiogenic embolism, such as history of atrial fibrillation; artery stenosis caused by dissection; hemorrhagic stroke; subarachnoid hemorrhage; moyamoya disease; fibromuscular dysplasia; arteriovenous malformation; aneurysm; signs of acute infection; tumor; hematological system disorder; severe liver and kidney function impairment. This study was approved by the Clinical Research Ethics Committee of Zhongnan Hospital of Wuhan University (Ref. No.:2,022,106 K).

### Clinical data collection and analysis

Basic clinical data, including gender, age, previous medical history (hypertension, diabetes mellitus, ischemic stroke), previous/current smoking, drinking, laboratory measurements within 24 h after admission (blood routine, lipid profile, fasting glucose) were collected from hospital information manage system. The TyG index was calculated by the following formula: TyG index = Ln [fasting glucose (mg/dL) * triglycerides (mg/dL)/2] [12].

### Imaging data collection and analysis

The DSA data of all patients were assessed independently by two neurointerventionists who have more than 5 years of experiences in interpreting DSA images; a third neurointerventionist confirmed the results when disagreements occurred. The definition of intracranial/extracranial arteries were as follows: Intracranial arteries included C6-C7 segments of internal carotid artery (ICA), M1-M2 segments of middle cerebral artery, A1-A2 segment of anterior cerebral artery, P1-P2 segment of posterior cerebral artery, V4 segment of vertebral artery, and basilar artery. Subclavian artery, V1-V3 segments of vertebral artery, common carotid artery, C1-C5 segments of ICA are classified as extracranial arteries. Patients were divided into ICAS group, ECAS group, or combined ICAS/ECAS group, when they present with only ICAS, only ECAS, or both. The degree of stenosis was assessed according to the Warfarin-Aspirin Symptomatic Intracranial Disease Study [13], which was calculated as follows: degree of stenosis (%) = (1-diameter at the narrowest point of the narrow segment/the diameter of the proximal normal vessel) × 100%. According to the degree of stenosis, the patients were divided into a mild stenosis group (stenosis degree of 49% or less), a moderate stenosis group (stenosis degree of 50–69%), and a severe stenosis group (stenosis degree of 70–99% or occlusion). Patients were considered to have stenosis when any degree of stenosis exists. Patients were assigned to symptomatic group in case of transient ischemic attack (TIA) and/or ischemic stroke in the territory of stenotic artery within the proceeding 1 month. Diagnosis of TIA and ischemic stroke were according to the AHA/ASA definition [14].

### Statistical analysis

Baseline characteristics were displayed according to the tertiles of TyG index, including age, sex, past medical history, blood routine test, lipid profile and fasting glucose. Continuous variables were presented as median with interquartile range (IQR) for non-Gaussian distribution, and as mean with standard deviation (SD) for Gaussian distribution; categorical variables were presented as proportions. Comparisons were performed by Kruskal-Wallis test, one-way ANOVA, and chi-square test, respectively.

The TyG index was analyzed as continuous variable and tertile (T1, T2 and T3) to investigate its association with arterial stenosis. Univariate and multivariable logistic regression were applied to evaluate the association between TyG index and arterial stenosis. Three models were applied: model 1, unadjusted; model 2, adjusted for age and sex; model 3, adjusted for age, sex, smoking, drinking, medical history of hypertension and stroke; model 4, adjusted for age, sex, smoking, drinking, medical history of hypertension and stroke, platelet, total cholesterol (TC), high-density lipoprotein (HDL), and low-density lipoprotein (LDL). Variables with multi-collinearity were not included in the model, including diabetes. Odds ratio (OR) with the 95% confidence interval (CI) was estimated. *p* for trend was applied to investigate the relationship of TyG index tertiles and arterial stenosis. Restricted cubic splines were constructed to flexibly model and visualize the non-linear relationship between TyG index and different arterial stenosis status. *p* values were two-sided and *p* < 0.05 was chosen as the significance level. All the statistical analyses were performed using statistical software R (version 4.2.3; R Foundation for Statistical Computing, Vienna, Austria).

## Results

### Baseline characteristics

**Fig. 1:**
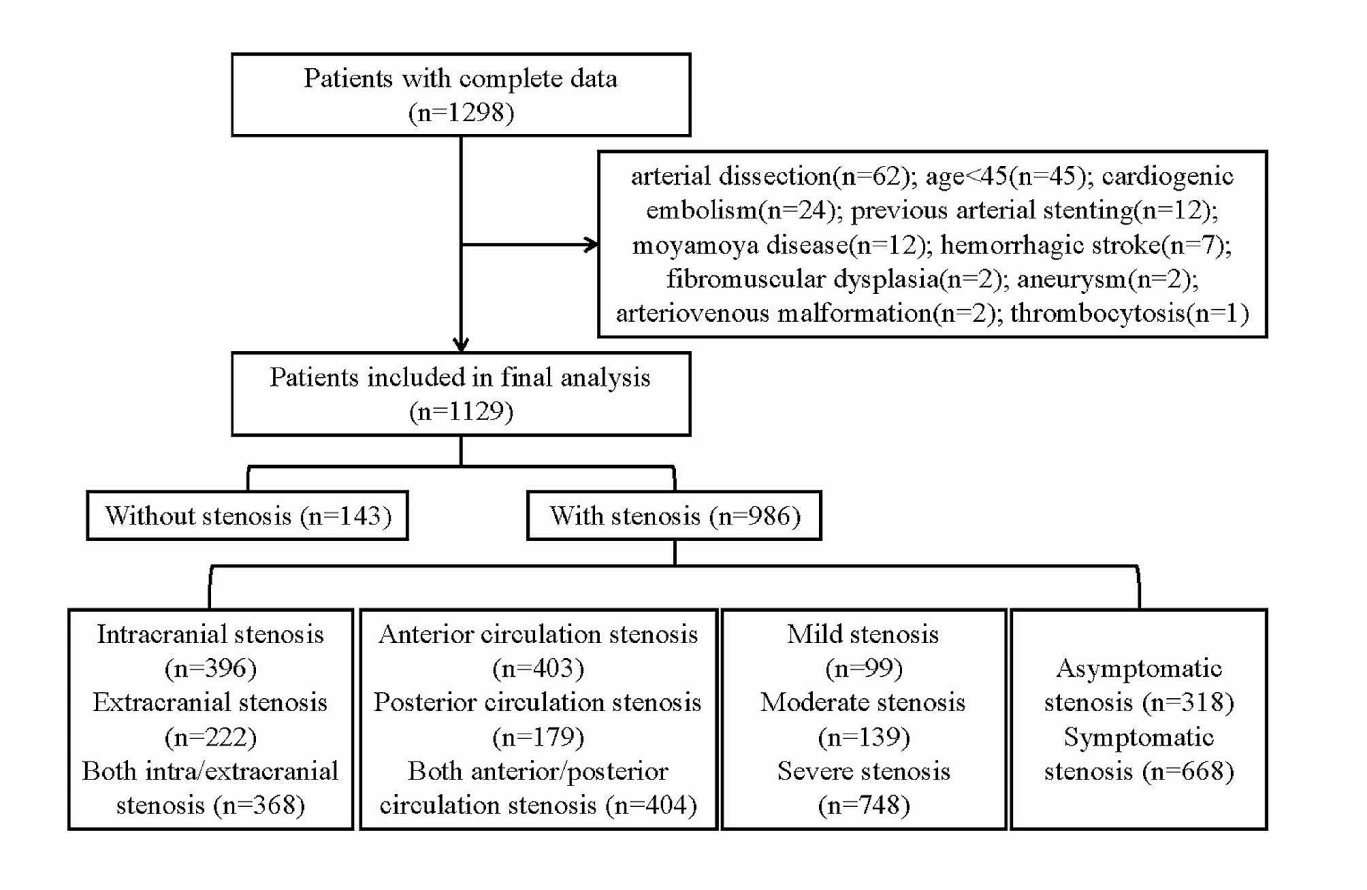
Flowchart

**Fig. 2:**
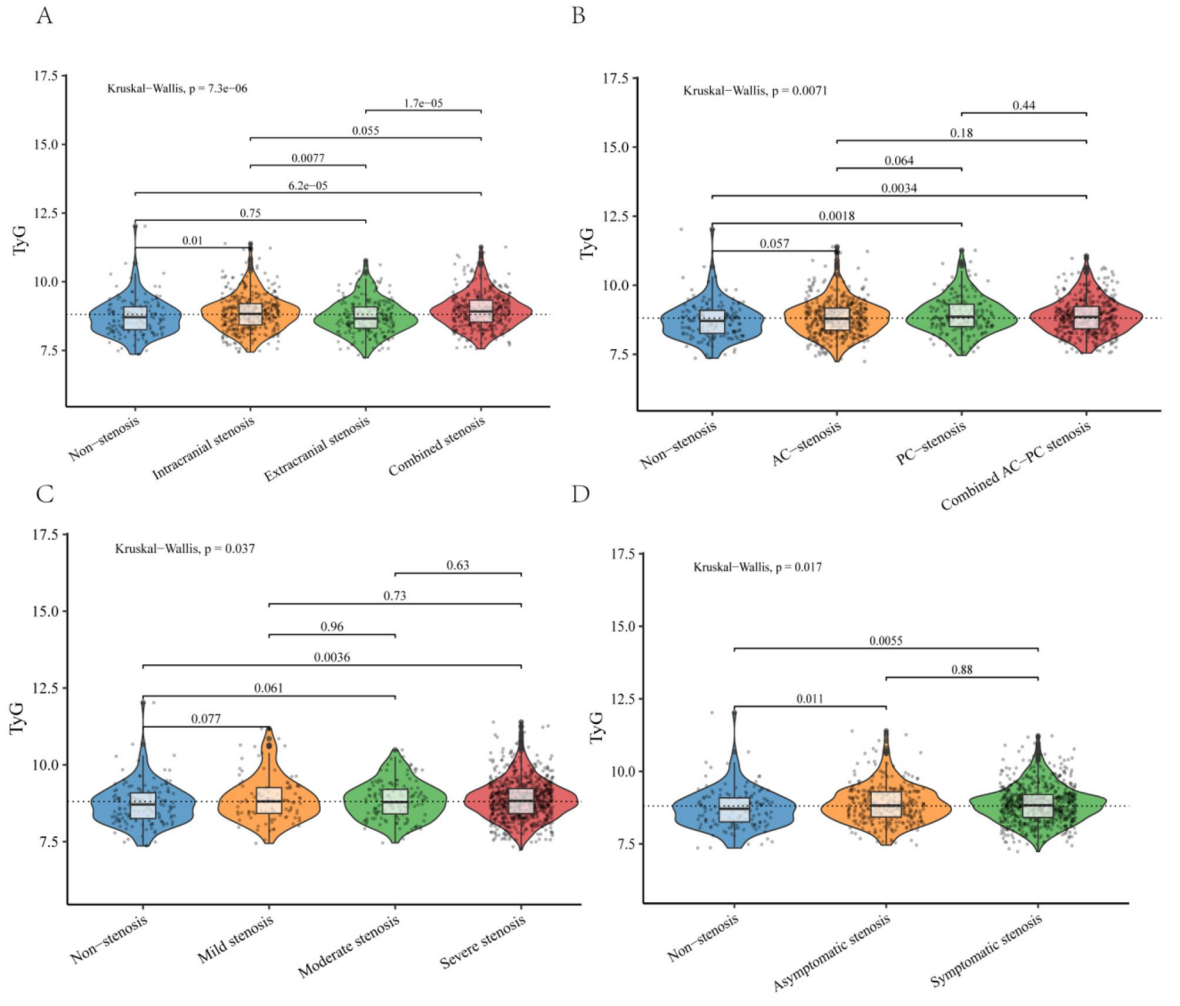
The violin plots demonstrating the distribution of the TyG index among patients in different groups: **A**: without stenosis, intracranial stenosis, extracranial stenosis, and combined intracranial and extracranial stenosis. **B**: without stenosis, anterior circulation (AC) stenosis, posterior circulation (PC) stenosis and combined anterior/posterior (AC-PC) stenosis. **C**: without stenosis, mild stenosis, moderate stenosis, and severe stenosis. **D**: without stenosis, asymptomatic stenosis, and symptomatic stenosis. TyG index: Triglyceride-glucose index

In total, there were 1298 patients with complete cerebrovascular DSA data and clinical data, and 1129 patients were finally recruited in our analysis (Fig. 1). The demographic and clinical characteristics of the included patients were displayed according to the tertiles of TyG index in Table 1. Among the enrolled patients, the median age was 62 (IQR 55–68) years, and 71.3% were male. The median fasting glucose was 5.61 (IQR 4.98–6.96) mmol/L, the median TyG index was 8.81 (IQR 8.40–9.21). The number of patients with ICAS, ECAS and combined ICAS/ECAS were 396, 222 and 368, respectively. The number of patients with anterior circulation stenosis, posterior circulation stenosis and combined anterior/posterior stenosis were 403, 179 and 404, respectively. 99 patients presented mild stenosis, 139 patients presented moderate stenosis, and 748 patients have severe stenosis. 318 patients had asymptomatic stenosis while 668 patients had symptomatic stenosis. The TyG index among patients with different arterial stenosis status was shown by violin plots in Fig. 2.

**Table 1:** Baseline characteristics of patients stratified by the TyG index tertiles

Characteristics	Total *n* = 1129	TyG index tertiles			*p*
		Q1(*n* = 376)	Q2(*n* = 376)	Q3(*n* = 377)	
General information					
Age [median (IQR)]	62 (55, 68)	64 (57, 70)	62 (55, 69)	60 (54, 65)	**< 0.001**
Male sex [n (%)]	805 (71.30%)	288 (76.60%)	265 (70.48%)	252 (66.84%)	**0.011**
Past medical history					
Hypertension [n (%)]	778 (68.91%)	243 (64.63%)	265 (70.48%)	270 (71.62%)	0.085
Diabetes [n (%)]	335 (29.67%)	56 (14.89%)	90 (23.94%)	189 (50.13%)	**< 0.001**
Stroke [n (%)]	271 (24.00%)	98 (26.06%)	87 (23.14%)	86 (22.81%)	0.516
Smoking [n (%)]	363 (32.15%)	123 (32.71%)	122 (32.45%)	118 (31.30%)	0.907
Drinking [n (%)]	153 (13.55%)	44 (11.70%)	53 (14.10%)	56 (14.85%)	0.848
Laboratory tests					
WBC, median (IQR), 10^9^/L	6.36 (5.17, 7.68)	6.00 (5.00, 7.30)	6.42 (5.14, 7.66)	6.62 (5.43, 8.16)	**< 0.001**
Platelet, median (IQR), 10^9^/L	199.00 (165.00, 237.00)	191.00 (163.00, 225.25)	206.00 (166.00, 243.25)	201.00 (167.00, 240.00)	**0.003**
TC, mean (SD) mmol/L	4.15 (1.12)	3.80 (0.94)	4.11 (1.01)	4.54 (1.25)	**< 0.001**
TG, median (IQR), mmol/L	1.40 (0.99, 1.89)	0.90 (0.74, 1.07)	1.48 (1.29, 1.70)	2.19 (1.74, 2.83)	**< 0.001**
HDL, mean (SD), mmol/L	1.02 (0.25)	1.12 (0.26)	1.01 (0.23)	0.93 (0.21)	**< 0.001**
LDL, mean (SD), mmol/L	2.55 (0.90)	2.33 (0.80)	2.59 (0.85)	2.72 (0.99)	**< 0.001**
GLU, median (IQR), mmol/L	5.61 (4.98, 6.96)	5.02 (4.59, 5.56)	5.59 (5.06, 6.47)	7.37 (5.76, 9.87)	**< 0.001**
TyG index	8.81 (8.40, 9.21)	8.23 (8.01, 8.40)	8.81 (8.68, 8.94)	9.41 (9.21, 9.80)	**< 0.001**
Stenosis location					**< 0.001**
No stenosis [n (%)]	143 (12.67%)	61 (16.22%)	45 (11.97%)	37 (9.81%)	
ICAS [n (%)]	396 (35.08%)	126 (33.51%)	132 (35.11%)	138 (36.60%)	
ECAS [n (%)]	222 (19.66%)	93 (24.73%)	71 (18.88%)	58 (15.38%)	
Combined ICAS/ECAS [n (%)]	368 (32.60%)	96 (25.53%)	128 (34.04%)	144 (38.20%)	
Stenosis distribution					0.110
No stenosis [n (%)]	143 (12.67%)	61 (16.22%)	45 (11.97%)	37 (9.81%)	
Anterior circulation [n (%)]	403 (35.70%)	138 (36.70%)	134 (35.64%)	131 (34.75%)	
Posterior circulation [n (%)]	179 (15.85%)	51 (13.56%)	58 (15.43%)	70 (18.57%)	
Combined anterior/posterior circulation [n (%)]	404 (35.78%)	126 (33.51%)	139 (36.97%)	139 (36.87%)	
Stenosis severity					0.118
No stenosis [n (%)]	143 (12.67%)	61 (16.22%)	45 (11.97%)	37 (9.81%)	
Mild stenosis [n (%)]	199 (8.77%)	138 (36.70%)	30 (7.98%)	31 (8.22%)	
Moderate stenosis [n (%)]	139 (12.31%)	51 (13.56%)	46 (12.23%)	45 (11.94%)	
Severe stenosis [n (%)]	748 (66.25%)	126 (33.51%)	255 (67.82%)	264 (70.03%)	
Stenosis symptom					0.098
No stenosis [n (%)]	143 (12.67%)	61 (16.22%)	45 (11.97%)	37 (9.81%)	
Asymptomatic [n (%)]	318 (28.17%)	106 (28.19%)	102 (27.13%)	110 (29.18%)	
Symptomatic [n (%)]	668 (59.17%)	209 (55.59%)	229 (60.90%)	230 (61.01%)	

WBC white blood cell, TC total cholesterol, TG triglyceride, HDL high-density lipoprotein, LDL low-density lipoprotein, GLU fasting glucose, TyG index Triglyceride-glucose index, ICAS intracranial atherosclerotic stenosis, ECAS extracranial atherosclerotic stenosis, IQR interquartile range. *p* for comparisons between ICAS, ECAS and combined ICAS/ECAS. Bold means that the *p* value is significant

### The association of TyG index and location of stenosis

Compared to patients without stenosis, elevated TyG index was associated with the presence of ICAS (OR, 1.45; 95%CI, 1.07–1.98, *p* = 0.017), and combined ICAS/ECAS (OR, 1.86; 95%CI, 1.34–2.58, *p* < 0.001). When the TyG index was analyzed by tertiles, compared with the first tertile, the third tertile of TyG were associated with increased odds of the presence of ICAS (*p* for trend = 0.014), the second and the third tertile of TyG were associated with increased odds of combined ICAS/ECAS (*p* for trend < 0.001). These associations were maintained after adjusting for age, sex, smoking, drinking, medical history of hypertension, stroke, platelet, TC, HDL, and LDL. However, TyG index was not associated with the presence of ECAS alone. Results were presented in Table 2.

**Fig. 3:**
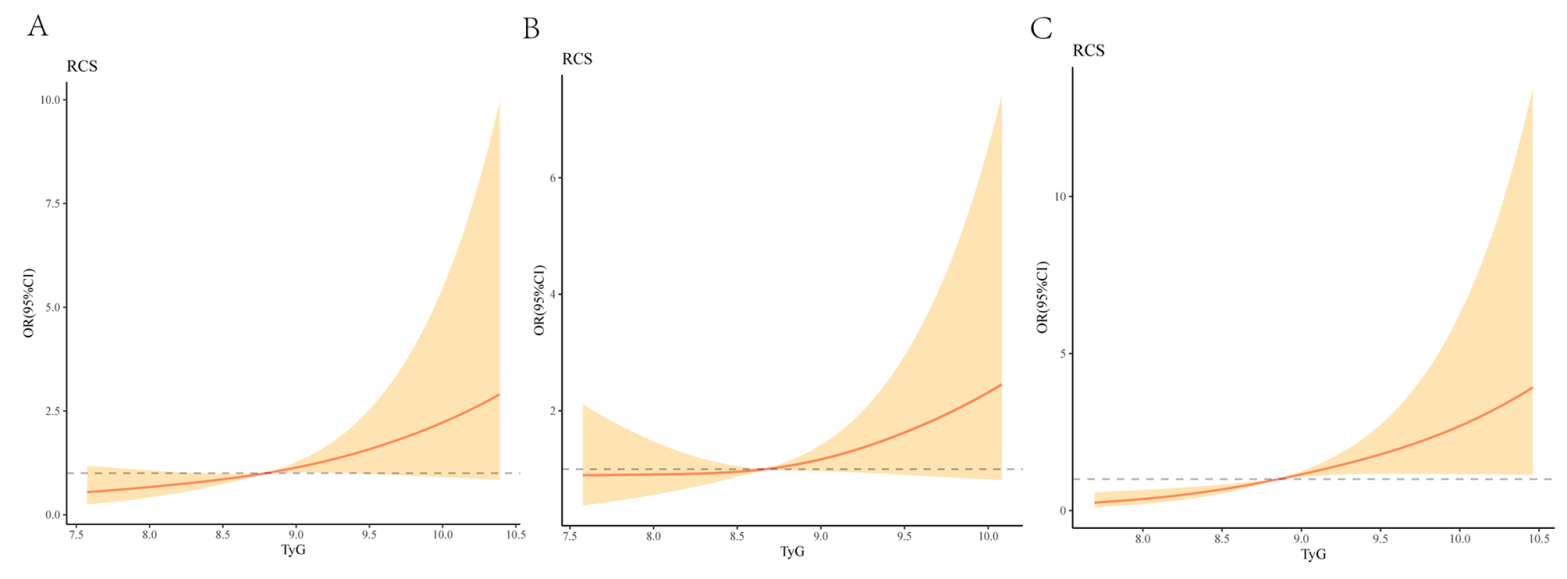
Association of TyG with the location of atherosclerotic stenosis Multivariable-adjusted ORs for the presence of intracranial(**A**), extracranial (**B**) and combined intracranial/extracranial stenosis (**C**) based on restricted cubic spines with 3 knots of TyG index. All models adjusted for age, sex, smoking, drinking, medical history of stroke, hypertension, platelet, TC, HDL, and LDL. TyG, Triglyceride glucose index

In Fig. 3, multivariable-adjusted spline regression models showed that a progressively increasing risk of the presence of ICAS, ECAS and combined ICAS/ECAS was related to an elevated TyG index. However, the relationship in patients with only ECAS was quite flat until TyG index of about 8.67, and then started to increase afterwards.

**Table 2:** Association of the TyG index with atherosclerotic stenosis in intracranial, extracranial, and combined intra/extracranial arteries

		Model 1^*^OR (95% CI)	*p*	Model 2^†^ OR (95% CI)	*p*	Model 3^#^ OR (95% CI)	*p*	Model 4^&^ OR (95% CI)	*p*
**Intracranial stenosis (n = 396)**									
TyG index		1.45(1.07–1.98)	0.017	1.48(1.09–2.03)	0.013	1.44(1.05–1.98)	0.025	1.77 (1.10–2.84)	0.019
TyG index tertiles	T1 (*n* = 126)	reference		reference		reference		reference	
	T2 (*n* = 132)	1.42(0.90–2.24)	0.132	1.44(0.91–2.28)	0.116	1.42(0.89–2.26)	0.139	1.37(0.84–2.23)	0.211
	T3 (*n* = 138)	1.81(1.12–2.90)	0.015	1.87(1.16–3.01)	0.011	1.80(1.11–2.94)	0.018	1.88(1.01–1.86)	0.046
	p for trend	1.35(1.06–1.71)	0.014	1.37(1.08–1.74)	0.010	1.35(1.06–1.72)	0.017	1.37(1.01–1.86)	0.044
**Extracranial stenosis (n = 222)**									
TyG index		1.03(0.74–1.43)	0.872	1.22(0.86–1.73)	0.260	1.20(0.84–1.71)	0.323	1.45(0.86–2.45)	0.166
TyG index tertiles	T1 (*n* = 93)	reference		reference		reference		reference	
	T2 (*n* = 71)	1.03(0.63–1.70)	0.892	1.19(0.70–2.02)	0.516	1.13(0.66–1.93)	0.651	1.13(0.64-2.00)	0.674
	T3 (*n* = 58)	1.03(0.61–1.74)	0.917	1.37(0.78–2.41)	0.272	1.34(0.75–2.37)	0.321	1.50(0.73–3.07)	0.273
	p for trend	1.02(0.78–1.32)	0.907	1.17(0.89–1.55)	0.265	1.15(0.87–1.53)	0.322	1.21(0.85–1.73)	0.287
**Combined intra/extracranial stenosis (n = 368)**									
TyG index		1.86 (1.34–2.58)	< 0.001	2.47 (1.72–3.54)	< 0.001	2.37 (1.64–3.43)	< 0.001	2.81(1.67–4.72)	< 0.001
TyG index tertiles	T1 (*n* = 96)	reference		reference		reference		reference	
	T2 (*n* = 128)	1.81 (1.13–2.88)	0.013	2.47 (1.49–4.11)	< 0.001	2.37 (1.42–3.98)	0.001	1.96(1.13–3.38)	0.017
	T3 (*n* = 144)	2.47 (1.53–4.01)	< 0.001	3.83 (2.26–6.51)	< 0.001	3.68 (2.14–6.34)	< 0.001	3.27(1.66–6.46)	0.001
	p for trend	1.58 (1.24–2.02)	< 0.001	1.97 (1.50–2.57)	< 0.001	1.93 (1.46–2.54)	< 0.001	1.82(1.29–2.56)	0.001

TyG index Triglyceride-glucose index

T1, T2, T3 indicate the 1st, 2nd, and 3rd tertile of TyG

^*^ Model 1: Non adjusted

^†^ Model 2: adjusted for age and sex

^#^ Model 3: adjusted for age, sex, smoking, drinking, medical history of hypertension, stroke

^&^Model 4: adjusted for age, sex, smoking, drinking, medical history of hypertension, stroke, platelet, TC, HDL, and LDL

### The association of TyG index and stenosis distribution

Compared to patients without stenosis, elevated TyG index was associated with the presence of posterior circulation stenosis (OR, 1.68; 95%CI, 1.18–2.39, *p* = 0.004), and combined anterior/posterior circulation stenosis (OR, 1.55; 95%CI, 1.13–2.13, *p* = 0.006). When the TyG index was analyzed by tertiles, compared with the first tertile, the third tertile of TyG index were associated with increased odds of the presence of posterior circulation stenosis (*p* for trend = 0.003) and combined anterior/posterior circulation stenosis (*p* for trend = 0.012). These associations were maintained after adjusting for age, sex, smoking, drinking, medical history of hypertension and stroke. The significant association of TyG index and anterior circulation stenosis was observed only when TyG index was analyzed by tertiles. Results were presented in Table 3.

**Fig. 4:**
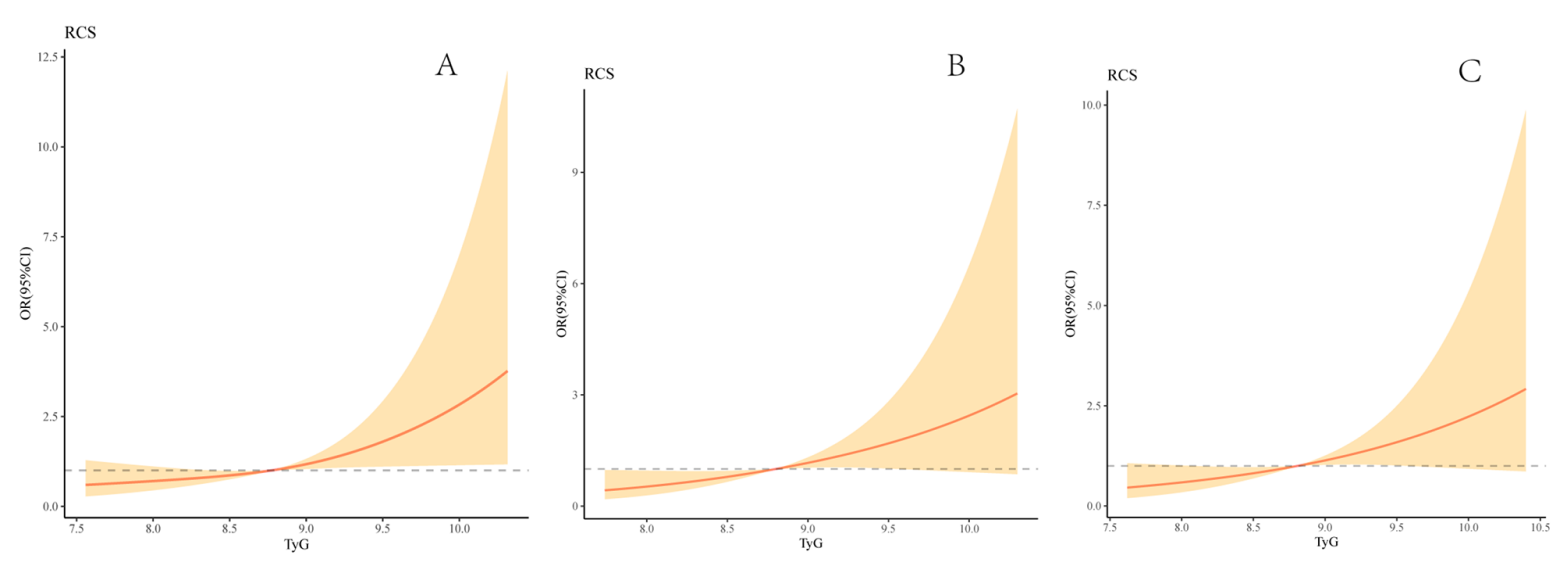
Association of TyG with the distribution of atherosclerotic stenosis Multivariable-adjusted ORs for the presence of anterior circulation (**A**), posterior circulation (**B**) and combined anterior/posterior circulation stenosis (**C**) based on restricted cubic spines with 3 knots of TyG index. All models adjusted for age, sex, smoking, drinking, medical history of stroke, hypertension, platelet, TC, HDL, and LDL. TyG, Triglyceride glucose index

In Fig. 4, multivariable-adjusted spline regression models showed that a progressively increasing risk of the presence of anterior circulation stenosis, posterior circulation stenosis and combined anterior/posterior circulation stenosis was related to an elevated TyG index.

**Table 3:** Association of the TyG index with stenosis distribution

		Model 1^*^OR (95% CI)	*p*	Model 2^†^ OR (95% CI)	*p*	Model 3^#^ OR (95% CI)	*p*	Model 4^&^ OR (95% CI)	*p*
**Anterior circulation stenosis (n = 403)**									
TyG index		1.30(0.96–1.75)	0.093	1.34(0.98–1.81)	0.064	1.35(0.99–1.85)	0.057	1.84(0.95–2.93)	0.059
TyG index tertiles	T1 (*n* = 61)	reference		reference		reference		reference	
	T2 (*n* = 45)	1.32(0.84–2.07)	0.234	1.35(0.86–2.13)	0.195	1.38 (0.87–2.19)	0.167	1.36(0.83–2.21)	0.219
	T3 (*n* = 37)	1.57(0.97–2.51)	0.064	1.64(1.01–2.64)	0.043	1.67(1.03–2.71)	0.038	1.89(1.03–3.47)	0.039
	p for trend	1.26(0.99–1.59)	0.059	1.28(1.01–1.63)	0.040	1.30(1.02–1.65)	0.035	1.37(1.02–1.85)	0.038
**Posterior circulation stenosis (n = 179)**									
TyG index		1.68(1.18–2.39)	0.004	1.87(1.29–2.72)	0.001	1.78(1.22–2.60)	0.003	2.17(1.23–3.84)	0.008
TyG index tertiles	T1 (*n* = 138)	reference		reference		reference		reference	
	T2 (*n* = 134)	1.54(0.90–2.64)	0.115	1.82(1.04–3.19)	0.036	1.64(0.93–2.91)	0.089	1.56(0.86–2.83)	0.144
	T3 (*n* = 131)	2.26(1.31–3.90)	0.003	2.78(1.57–4.94)	< 0.001	2.59(1.44–4.65)	0.002	2.67(1.27–5.63)	0.010
	p for trend	1.51(1.15–1.98)	0.003	1.67(1.25–2.23)	< 0.001	1.61(1.20–2.16)	0.002	1.63(1.12–2.36)	0.010
**Combined anterior/posterior circulation stenosis (n = 404)**									
TyG index		1.55 (1.13–2.13)	0.006	2.06 (1.46–2.90)	< 0.001	1.87 (1.32–2.67)	< 0.001	1.94(1.17–3.22)	0.010
TyG index tertiles	T1 (*n* = 126)	reference		reference		reference		reference	
	T2 (*n* = 139)	1.50(0.95–2.36)	0.083	1.86 (1.14–3.02)	0.013	1.69 (1.02–2.80)	0.041	1.40(0.81–2.39)	0.224
	T3 (*n* = 139)	1.82 (1.13–2.92)	0.013	2.73 (1.63–4.57)	< 0.001	2.41 (1.43–4.18)	0.001	1.94(0.98–3.81)	0.055
	p for trend	1.36 (1.07–1.72)	0.012	1.66(1.28–2.15)	< 0.001	1.57 (1.20–2.05)	0.001	1.39(0.99–1.95)	0.054

TyG index Triglyceride-glucose index

T1, T2, T3 indicate the 1st, 2nd, and 3rd tertile of TyG

^*^ Model 1: Non adjusted

^†^ Model 2: adjusted for age and sex

^#^ Model 3: adjusted for age, sex, smoking, drinking, medical history of hypertension, stroke

^&^Model 4: adjusted for age, sex, smoking, drinking, medical history of hypertension, stroke, platelet, TC, HDL, and LDL

### The association of TyG index and stenosis severity

As presented in Table 4, elevated TyG index was associated with the presence of severe stenosis (OR, 1.49; 95%CI, 1.11–1.99, *p* = 0.008). When the TyG index was analyzed by tertiles, compared with the first tertile, the second and the third tertile of TyG were associated with increased odds of the presence of severe stenosis (*p* for trend = 0.004). These associations were maintained after adjusting for potential covariates (age, sex, smoking, drinking, medical history of hypertension and stroke, platelet, TC, HDL, and LDL). However, the association of TyG index and mild or moderate stenosis was not established.

**Fig. 5:**
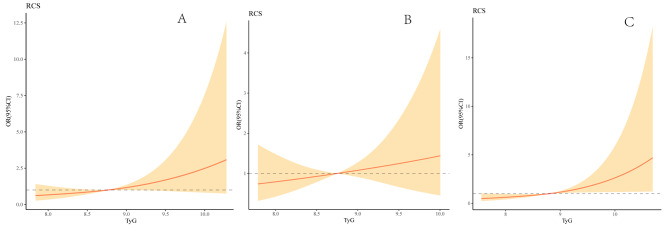
Association of TyG with the stenosis severity Multivariable-adjusted ORs for the presence of mild (**A**), moderate (**B**) and severe stenosis (**C**) based on restricted cubic spines with 3 knots of TyG index. All models adjusted for age, sex, smoking, drinking, medical history of stroke, hypertension, platelet, TC, HDL, and LDL. TyG, Triglyceride glucose index

In Fig. 5, multivariable-adjusted spline regression models showed that a progressively increasing risk of the presence of mild, moderate, and severe stenosis was related to an elevated TyG index.

**Table 4:** Association of the TyG index with atherosclerotic stenosis severity

		Model 1^*^OR (95% CI)	*p*	Model 2^†^ OR (95% CI)	*p*	Model 3^#^ OR (95% CI)	*p*	Model 4^&^ OR (95% CI)	*p*
**Mild stenosis (n = 199)**									
TyG index		1.45(0.99–2.11)	0.054	1.64(1.10–2.45)	0.015	1.64(1.09–2.46)	0.017	1.88 (0.99–3.59)	0.055
TyG index tertiles	T1 (*n* = 138)	reference		reference		reference		reference	
	T2 (*n* = 30)	1.07(0.58–1.98)	0.829	1.22(0.64–2.33)	0.541	1.21(0.63–2.32)	0.057	0.97(0.49–1.95)	0.939
	T3 (*n* = 31)	1.34(0.72–2.52)	0.353	1.64(0.85–3.16)	0.143	1.63(0.84–3.17)	0.150	1.14(0.48–2.72)	0.766
	p for trend	1.15(0.85–1.58)	0.366	1.28(0.92–1.77)	0.146	1.27(0.91–1.77)	0.154	1.06(0.69–1.63)	0.797
**Moderate stenosis (n = 139)**									
TyG index		1.37(0.94-2.00)	0.099	1.54(1.04–2.28)	0.032	1.45(0.96–2.18)	0.076	1.35(0.73–2.50)	0.336
TyG index tertiles	T1 (*n* = 51)	reference		reference		reference		reference	
	T2 (*n* = 46)	1.30(0.74–2.27)	0.358	1.49(0.84–2.66)	0.174	1.38(0.76–2.49)	0.293	1.12(0.59–2.10)	0.732
	T3 (*n* = 45)	1.55(0.87–2.75)	0.139	1.89(1.04–3.46)	0.038	1.74(0. 93-3.28)	0.083	1.39(0.64–3.03)	0.408
	p for trend	1.25(0.93–1.66)	0.134	1.38(1.02–1.86)	0.036	1.32(0.97–1.81)	0.081	1.17(0.80–1.73)	0.416
**Severe stenosis (n = 748)**									
TyG index		1.49 (1.11–1.99)	0.008	1.66 (1.23–2.24)	0.001	1.59 (1.17–2.15)	0.003	1.97(1.27–3.05)	0.002
TyG index tertiles	T1 (*n* = 126)	reference		reference		reference		reference	
	T2 (*n* = 255)	1.51 (0.99–2.31)	0.057	1.66 (1.08–2.57)	0.021	1.61 (1.04–2.50)	0.033	1.54(0.97–2.45)	0.067
	T3 (*n* = 264)	1.90 (1.22–2.97)	0.005	2.27 (1.44–3.59)	< 0.001	2.18 (1.73–3.47)	0.001	2.35(1.30–4.23)	0.005
	p for trend	1.39 (1.11–1.73)	0.004	1.52 (1.21–1.91)	< 0.001	1.48 (1.17–1.87)	0.001	1.53(1.15–2.05)	0.004

TyG index Triglyceride-glucose index

T1, T2, T3 indicate the 1st, 2nd, and 3rd tertile of TyG

^*^ Model 1: Non adjusted

^†^ Model 2: adjusted for age and sex

^#^ Model 3: adjusted for age, sex, smoking, drinking, medical history of hypertension, stroke

^&^Model 4: adjusted for age, sex, smoking, drinking, medical history of hypertension, stroke, platelet, TC, HDL, and LDL

### The association of TyG index and asymptomatic/symptomatic stenosis

As presented in Table 5, elevated TyG index was associated with the presence of both asymptomatic stenosis (OR, 1.47; 95%CI, 1.07–2.03, *p* = 0.018) and symptomatic stenosis (OR, 1.46; 95%CI, 1.09–1.96, *p* = 0.011). When the TyG index was analyzed by tertiles, compared with the first tertile, the third tertile of TyG were associated with increased odds of the presence of both asymptomatic and symptomatic stenosis (*p* for trend = 0.030 and 0.008, respectively). These associations were maintained after adjusting for potential covariates (age, sex, smoking, drinking, medical history of hypertension and stroke, platelet, TC, HDL, and LDL).

**Fig. 6:**
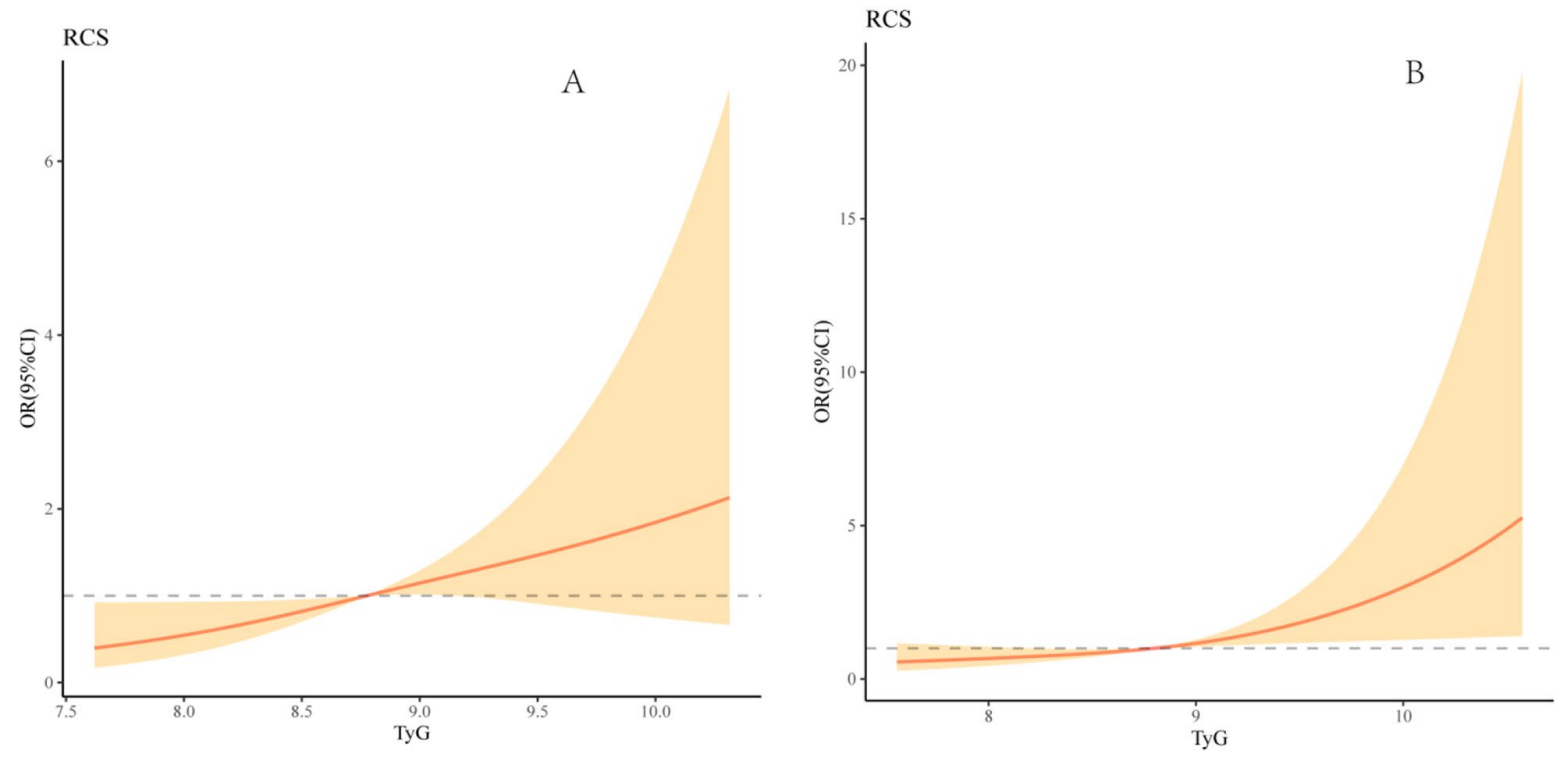
Association of TyG with the symptomatic and asymptomatic stenosis Multivariable-adjusted ORs for the presence of asymptomatic stenosis (**A**) and symptomatic stenosis (*B*) based on restricted cubic spines with 3 knots of TyG index. All models adjusted for age, sex, smoking, drinking, medical history of stroke, hypertension, platelet, TC, HDL, and LDL. TyG, Triglyceride glucose index

In Fig. 6, multivariable-adjusted spline regression models showed that a progressively increasing risk of the presence of asymptomatic and symptomatic stenosis was related to an elevated TyG index.

**Table 5:** Association of the TyG index with asymptomatic and symptomatic atherosclerotic stenosis

		Model 1^*^OR (95% CI)	*p*	Model 2^†^ OR (95% CI)	*p*	Model 3^#^ OR (95% CI)	*p*	Model 4^&^ OR (95% CI)	*p*
**Asymptomatic stenosis (n = 318)**									
TyG		1.47(1.07–2.03)	0.018	1.80(1.27–2.54)	0.001	1.63(1.14–2.32)	0.007	1.71(1.18–2.48)	0.004
TyG tertiles	T1 (*n* = 106)	reference		reference		reference		reference	
	T2 (*n* = 102)	1.30(0.81–2.09)	0.269	1.52(0.93–2.50)	0.095	1.37(0.83–2.28)	0.219	1.39(0.83–2.32)	0.208
	T3 (*n* = 110)	1.71(1.05–2.79)	0.031	2.31(1.37–3.88)	0.002	2.04(1.20–3.47)	0.009	2.15(1.24–3.71)	0.006
	p for trend	1.31(1.03–1.67)	0.030	1.52(1.17–1.97)	0.002	1.43(1.09–1.86)	0.009	1.46(1.11–1.92)	0.006
**Symptomatic stenosis (n = 668)**									
TyG		1.46(1.09–1.96)	0.011	1.57(1.16–2.11)	0.003	1.50(1.11–2.02)	0.008	1.48(1.09–2.01)	0.011
TyG tertiles	T1 (*n* = 209)	reference		reference		reference		reference	
	T2 (*n* = 229)	1.49(0.97–2.28)	0.070	1.60(1.03–2.47)	0.036	1.55(1.00-2.41)	0.052	1.52(0.97–2.37)	0.065
	T3 (*n* = 230)	1.81(1.16–2.84)	0.009	2.04(1.29–3.23)	0.002	1.95(1.23–3.10)	0.005	1.91(1.20–3.06)	0.007
	p for trend	1.36(1.08–1.70)	0.008	1.44(1.14–1.81)	0.002	1.40(1.11–1.77)	0.004	1.39(1.10–1.76)	0.006

TyG index Triglyceride-glucose index

T1, T2, T3 indicate the 1st, 2nd, and 3rd tertile of TyG

^*^ Model 1: Non adjusted

^†^ Model 2: adjusted for age and sex

^#^ Model 3: adjusted for age, sex, smoking, drinking, medical history of hypertension, stroke

^&^Model 4: adjusted for age, sex, smoking, drinking, medical history of hypertension, stroke, platelet, TC, HDL, and LDL

## Discussion

This retrospective study investigated the association of TyG index and ICAS/ECAS, which was evaluated by cerebrovascular DSA examination. Our findings demonstrated that elevated TyG index was related to higher presence of ICAS, combined ICAS/ECAS, anterior circulation stenosis, posterior circulation stenosis, combined anterior/posterior circulation stenosis, severe stenosis, both symptomatic and asymptomatic stenosis.

Insulin resistance was an important pathogenesis for type 2 diabetes mellitus and was related to atherosclerotic cardiovascular disease [15]. TyG index, a simple alternative surrogate of insulin resistance, have been suggested as a risk factor of cardiovascular and cerebrovascular disease in several studies [6, 16–20]. In a retrospective observational cohort study, patients with higher TyG index had a higher risk of stroke and myocardial infarction; therefore, TyG index may be helpful in early identification of high-risk population of cardiovascular event [16]. In a Chinese cohort study, 42,651 participants were followed up for 4.7 years, and the results revealed that 1-unit increase in the TyG index was associated with 1.16-fold higher risk in cardiovascular disease and with 1.39-fold higher risk in stroke [17]. A Rural Chinese cohort study followed 11,777 stroke-free general population for 6 years, and concluded that elevated TyG index might be an independent predictor of ischemic stroke [6]. Another prospective, multicenter cohort study showed that TyG was a significant risk factor for symptomatic intracranial atherosclerosis in hypertensive patients [19]. Wu Y et al. followed 4710 patients for 3 years, and concluded that a constant higher level of TyG index was associated with a higher risk of stroke [20].

Our findings indicated that TyG index was an indicator of ICAS, but not ECAS. The result is consistent with a Spain study, which demonstrated that insulin resistance emerged as an important molecular pathway involved in the development of intracranial atherosclerotic disease from its asymptomatic stage [21]. While other studies demonstrated different results with ours. Wang M et al. investigated participants without diabetes and concluded that elevated TyG index was associated with increased odds of atherosclerosis in both intra- and extracranial arteries [8]. Wang A et al. suggested an association of elevated TyG index and higher risk of ECAS, but not with ICAS [22]. The discrepancy could be explained by the difference in population selection and arterial stenosis measurement method. Wang M et al. applied CTA or MRA to measure arterial stenosis while DSA was used in ours [8]. Wang A et al. investigated asymptomatic healthy population, in addition, transcranial doppler (TCD) ultrasound and carotid duplex ultrasound were used to evaluate ICAS and ECAS [22].

The reason for the difference in correlation between the TyG index and ICAS and ECAS is still unclear. This may be due to the different structures and hemodynamics between intracranial and extracranial artery, and there are also differences in vascular risk factors between them [9]. A study suggested that intracranial arteries presented greater resistance to atherogenesis than extracranial arteries, and showed accelerated atherogenesis when antioxidant protection decreases with increasing age [23]. Furthermore, a study analyzed 933 subjects with moderate to high vascular risk, and showed that insulin resistance was an independent risk factor of intracranial atherosclerotic disease, but not extracranial pathology [21]. Another Chinses study enrolled 2007 residents aged ≥ 40 years and revealed that insulin resistance might predict the prevalence of ICAS in male participants [24]. Therefore, TyG index, as an indicator of insulin resistance, was associated with ICAS.

Our study demonstrated that TyG index was significantly associated with both anterior and posterior circulation stenosis, although the correlation with anterior circulation stenosis was not as strong. The disparity of anterior and posterior circulation stenosis was in accordance with previous studies. In a Chinese study, posterior circulation stroke patients presented higher prevalence of diabetes, higher TG, and lower HDL [25]. Another study found that diabetes was independently associated with an increase in the odds of posterior circulation ischemic stroke [26]. A Korean study revealed that compared with anterior circulation stenosis, posterior circulation stenosis was more often associated with diabetes and metabolic syndrome [10]. As an indicator of insulin resistance, the TyG index is also a marker of metabolic syndrome, with a stronger association with posterior circulation stenosis. However, the disparity of anterior and posterior circulation stenosis was unclear. Perhaps the neurovascular origin between anterior and posterior circulation differs, and the posterior circulation arteries might be more susceptible to metabolic disorders.

Only severe stenosis was significantly associated with the TyG index in our cohort, in accordance with previous studies [19]. Therefore, the TyG index may alert us to potential severe ICAS/ECAS in patients who have not undergone timely vascular assessment.

Our study found that both asymptomatic and symptomatic arterial stenosis were associated with increased TyG index, consist with many other studies [19, 21, 24]. However, our results failed to find out the difference of TyG level between asymptomatic and symptomatic stenosis, possibly due to the existence of other important factors determining whether stenosis is symptomatic, such as characteristics of atherosclerotic plaque, as well as relatively small sample size in our study.

This study has several limitations. First, we only investigated arterial stenosis as outcome variable, and did not consider other clinical features, such as whether the patient suffered acute ischemic stroke, medical treatment, neurological severity, and stroke recurrence. Second, due to the retrospective cross-sectional study design, we could not determine the causal relationship between the TyG index and arterial stenosis. Third, this is a single center study, our results should be verified in multicenter studies.

## Conclusion

In conclusion, the present study demonstrated that elevated TyG index was significantly associated with ICAS, combined ICAS/ECAS, anterior circulation stenosis, posterior circulation stenosis, combined anterior/posterior circulation stenosis, severe stenosis, both asymptomatic and symptomatic stenosis. TyG index might be a useful indicator of ICAS and severe stenosis. Future multicenter prospective studies are needed to validate our results.

## Data Availability

No datasets were generated or analysed during the current study.

## Abbreviations

ICAS: Intracranial atherosclerotic stenosis.
ECAS: Extracranial atherosclerotic stenosis
TyG index: Triglyceride-glucose index
CTA: Computed tomography angiography
MRA: Magnetic resonance angiography
DSA: Digital subtraction angiography
ICA: Internal carotid artery
TIA: Transient ischemic attack
IQR: Interquartile range
SD: Standard deviation
TC: Total cholesterol
TG: Triglyceride
HDL: High-density lipoprotein
LDL: Low-density lipoprotein
OR: Odds ratio
CI: Confidence interval
WBC: White blood cell
GLU: Fasting glucose
AC: Anterior circulation
PC: Posterior circulation
TCD: Transcranial doppler
